# Change in BMI after radioactive iodine ablation for graves disease

**DOI:** 10.1186/s13633-017-0044-z

**Published:** 2017-06-02

**Authors:** Melinda Chen, Matthew Lash, Todd Nebesio, Erica Eugster

**Affiliations:** 1 Department of Pediatrics, Section of Pediatric Endocrinology, Riley Hospital for Children, Indiana University School of Medicine, 705 Riley Hospital Drive, Room # 5960, Indianapolis, IN 46202 USA; 2 Department of Pediatrics, Naval Medical Center, 34800 Bob Wilson Dr, San Diego, CA 92134 USA

**Keywords:** RAI, Obesity, Graves disease

## Abstract

**Background:**

We aimed to determine the extent of post-treatment weight gain that occurs in pediatric patients in the first year following radioactive iodine (RAI) therapy for Graves disease (GD) and its relationship to clinical characteristics.

**Methods:**

A retrospective chart review of patients receiving RAI therapy for GD between 1998–2015 was performed. Change in BMI SDS (∆BMI SDS) from baseline to one year after treatment was determined. We also investigated whether individual clinical and/or biochemical factors were associated with the weight trajectory in these patients.

**Results:**

One hundred fifty seven patients aged 12.7 ± 3 years (80% girls) were included in the analysis. Average ∆BMI SDS was 0.70 ± 0.71 (*p <* 0.001) at 1 year. Patients with weight loss at presentation had a greater ∆BMI SDS than those without (0.92 vs 0.56, *p =* 0.005), whereas no association was seen with gender, pubertal status, use of antithyroid drugs, history of ADHD, or Down syndrome. Baseline BMI SDS was negatively correlated with ∆BMI SDS, with a stronger correlation in males. From baseline to 1 year, the proportion of overweight and obese patients increased from 9.6% to 18.5% and from 6.4% to 21%, respectively. In a subset of 81 patients, a positive correlation was noted between time to euthyroidism and ∆BMI SDS, particularly in boys.

**Conclusions:**

The number of our patients in the overweight category doubled and the number in the obese category more than tripled in the first year following RAI treatment for GD. Anticipatory guidance regarding this important issue is badly needed.

## Background

Graves disease (GD), characterized by autoimmune overstimulation of the thyroid gland, is the most common cause of hyperthyroidism in children and adolescents [1–4]. The overall incidence within the pediatric population is approximately 1 in 10,000 [5] with the highest prevalence seen in adolescent girls [2]. Although the hypermetabolic state in GD typically results in weight loss, stable weight or even weight gain due to increased caloric intake can be seen [5].

Therapeutic modalities for the treatment of GD include surgery, radioactive iodine (RAI) ablation and anti-thyroid drugs (ATDs). Surgery and RAI are considered definitive therapy and result in permanent hypothyroidism. Patients receiving definitive therapy often become hypothyroid for a period of time before appropriate levothyroxine replacement and biochemical euthyroidism is achieved. Weight gain following treatment of GD has been reported in both children and adults. Proposed explanations include continued increased caloric consumption with resolution of the prior hypermetabolic state [5], a return to or exaggeration of premorbid weight [6], or a result of post-treatment hypothyroidism [7]. Previous studies reporting change in weight following treatment of GD in children have focused primarily on patients treated with ATDs as first-line therapy. In contrast, the change in BMI after RAI treatment has not been systematically studied. It is unknown whether the treatment modality itself affects the degree of weight gain or whether there are clinical or biochemical factors that might modulate the degree of weight gain in these patients [7–9].

Thus, the goal of our study was to investigate the degree of weight gain in the first year following RAI therapy for GD. We also sought to explore whether individual characteristics were associated with differences in weight gain among our patients.

## Methods

Following ethical review and approval of Protocol #1412088318 by the Indiana University Institutional Review Board, a retrospective chart review was performed of pediatric patients receiving RAI ablation for GD at Riley Hospital for Children between 1998 and 2015. All patients with a documented height and weight within four months before RAI treatment and a follow up visit with documented height and weight between 6 and 18 months after RAI date were included. Regardless of whether patients had been treated for GD prior to evaluation at our institution, all patients were hyperthyroid at the time of RAI. If a second dose of RAI was needed, the dose leading to successful ablation (i.e. the second dose) was used as the event of interest to determine pre- and post-treatment height and weight.

Medical records were searched for variables of interest, which included age, gender, pubertal status, history of weight loss, DS (Down Syndrome), use of medications for ADHD, ATD use for ≥ one month prior to RAI, start date of levothyroxine replacement, and the date of documented euthyroidism. Weight and height were collected at baseline (last appointment prior to RAI) and at the one-year follow up appointment. BMI was calculated using the formula {BMI = weight in kilograms/height in meters^2^}. Pubertal status was classified as either pre-pubertal (Tanner I) or pubertal (Tanner II-V). Euthyroidism was considered to be a TSH and free T4 or total T4 within the performing laboratory’s reference range, or (in a small subset of cases), documented interpretation of lab tests as “normal” by the primary endocrinologist.

All BMIs were categorized according to Centers for Disease Control (CDC) standards as underweight (<5^th^ percentile), normal weight (5- < 85^th^ percentile), overweight (85- < 95^th^ percentile) or obese (≥95^th^ percentile) [10]. Time to euthyroidism (TTE) was calculated by determining the time in weeks between the date of starting levothyroxine replacement and the date of documented euthyroidism.

### Statistics

Analyses were performed using SPSS statistical software (version 24; IBM Corp.). All variables of interest were evaluated for normality to determine appropriateness of statistical methods and found to be normally distributed. Descriptive statistics were used to report anthropometric data and time intervals. Chi-square tests and Fisher’s exact tests were used to analyze differences in weight status category distribution before and after RAI. Paired t-tests were used to assess differences in BMI before and after treatment. Unpaired t-tests were used to analyze the effect of clinical risk factors on weight gain. Linear regression was used to describe strength of association between change (∆) in BMI SDS over time and contributing factors. The level of significance was α = 0.05, with all p-values lower than 0.05 considered statistically significant. Results are expressed as mean ± SD in the text, with medians and ranges also provided in Table 1.

**Table 1:** Potential factors associated with weight gain in patients following RAI treatment of GD

Variable – Mean ± SD Median (range)	Whole population (*n =* 157)			Sub-analysis (*n =* 81)		
Age	12.68 ± 3.00 y			12.79 ± 3.02 y		
	12.86 (5.34-17.89) y			12.77 (5.34-17.83) y		
	∆BMI SDS		*p*-value	∆BMI SDS		*p*-value
Gender	Female (%) 0.71 ± 0.66 (79.6)	Male (%) 0.71 ± 0.92 (20.4)	0.99	Female (%) 0.69 ± 0.65 (85.2)	Male (%) 0.35 ± 0.47 (14.8)	0.09
	0.61 (−0.4-2.97)	0.41 (−0.55-3.85)		0.69 (−0.4-2.68)	0.24 (−0.55-1.24)	
	Yes (%)	No (%)		Yes (%)	No (%)	
ATD use	0.64 ± 0.68 (21)	0.73 ± 0.73 (79)	0.55	0.67 ± 0.77 (18.5)	0.63 ± 0.60 (81.5)	0.81
	0.46 (−0.37-2.68)	0.54 (−0.55-3.85)		0.55 (−0.25-2.68)	0.66 (−0.55-2.04)	
Down syndrome	0.85 ± 0.86 (6.4)	0.70 ± 0.71 (93.6)	0.53	1.04 ± 0.90 (7.4)	0.61 ± 0.60 (92.6)	0.10
	0.88 (0.0-2.68)	0.53 (−0.55-3.85)		0.88 (0.17-2.68)	0.59 (−0.55-2.04)	
ADHD treatment	0.97 ± 1.08 (7.6)	0.69 ± 0.68 (92.4)	0.40	0.98 ± 0.79 (4.9)	0.62 ± 0.62 (95.1)	0.26
	0.44 (0.08-3.85)	0.55 (−0.55-2.97)		0.97 (0.08-1.92)	0.59 (−0.55-2.68)	
History of weight loss	0.92 ± 0.84 (38.2)	0.56 ± 0.57 (49)	0.005*	0.77 ± 0.62 (39.5)	0.55 ± 0.56 (50.6)	0.12
	0.74 (−0.55-3.85)	0.45 (−0.4-2.47)		0.74 (−0.55-2.00)	0.57 (−0.4-2.04)	
Pubertal	0.67 ± 0.63 (56.1)	0.91 ± 0.90 (29.9)	0.10	0.57 ± 0.58 (56.8)	0.90 ± 0.71 (29.6)	0.04*
	0.49 (−0.4-2.71)	0.75 (−0.37-3.85)		0.50 (−0.4-2.04)	0.82 (−0.35-2.68)	

Significant findings (*p <* 0.05) are marked with an asterisk (*). Mean ± SD are on line 1 and median, range on line 2 for all variables. Not all percentages add to 100% due to missing patient information. BMI = body mass index; ATD = anti-thyroid drug; ADHD = attention deficit-hyperactivity disorder

## Results

Of 247 patients receiving RAI for GD, 157 (79.6% female) had complete data and were included in the analysis. Mean baseline age was 12.7 ± 3.0 years and mean baseline BMI SDS was 0.003 ± 1.18 kg/m^2^. Ten patients (6.4%) had DS (Down Syndrome) and 33 (21.0%) had received ATDs before RAI ablation. Of 135 patients with documented pubertal status, 88 (65.2%) were ≥ Tanner stage II at the baseline evaluation. Post-treatment BMI data were obtained at 50.65 ± 11.32 weeks after RAI.

Average ∆BMI SDS from baseline to one year was 0.70 ± 0.71 (*p <* 0.001), with no difference noted by gender, pubertal status, history of ATDs, use of stimulant medications for ADHD, or presence of DS (Down Syndrome). In contrast, BMI SDS increased more in those with a history of weight loss (∆BMI SDS 0.92 vs 0.56, *p =* 0.005). These results are shown in Table 1. On regression analysis, baseline BMI SDS was negatively correlated with ∆BMI SDS (*p <* 0.001, R = −0.529), with a stronger correlation in males (*p <* 0.001, R = −0.696).

**Fig. 1:**
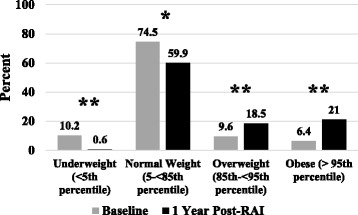
BMI classification of patients with Graves disease before and 1 year after RAI treatment. * = *p <* 0.01; ** = *p <* 0.001

From baseline to follow-up, the proportion of underweight patients in the study decreased from 10.2% to 0.6% (*p ≤* 0.001), while those in the overweight and obese categories increased from 9.6% to 18.5% (*p ≤* 0.001) and from 6.4% to 21% (*p ≤* 0.001), respectively, as shown in Fig. 1. Of 157 patients, 2 (0.1%) moved into a lower weight category, while 62 (39.5%) moved into a higher weight category. Notably, nearly one third of our patients (31.2%) moved into the overweight or obese category from a lower weight category.

**Fig. 2:**
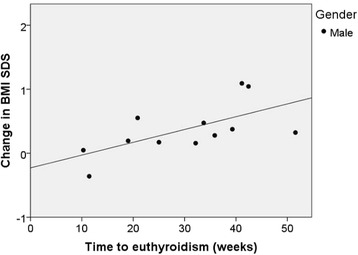
Relationship of ∆BMI SDS to time to euthyroidism in boys

A sub-analysis was performed on 81 patients (85.2% female) with evidence of good compliance in whom the exact TTE was available. Within this group, TTE was 24.16 ± 13.28 weeks and ∆BMI SDS at one year was not different from the group as a whole*.* Unlike what was seen for the entire cohort, a history of weight loss had no impact on ∆BMI SDS by 1 year, (0.77 ± 0.62 vs. 0.55 ± 0.56, *p =* 0.12), whereas prepubertal patients were found to have greater ∆BMI SDS than pubertal patients at 1 year (0.90 ± .71 vs 0.57 ± 0.58, *p =* 0.037). In males, a longer TTE was correlated with greater increase in BMI z-score (R = 0.63, *p =* 0.029) (Fig. 2), while a similar, though weaker, association was seen in females (R = 0.59, *p ≤* 0.001).

## Discussion

Weight gain after treatment for GD is well recognized in adults, but only two previous studies have been published in pediatric patients to our knowledge [5, 11]. These studies included patients who were almost exclusively treated with ATDs as first-line therapy, and did not closely examine as many potential modifying factors. We observed a ∆BMI SDS of 0.70 ± 0.71 over the first year in our population receiving RAI therapy as definitive treatment for GD. One previous study followed children for up to 3 years and observed a similar increase in BMI z-score after treatment from −0.02 ± 1.05 to 0.79 ± 0.81 with subsequent stabilization of weight, with most weight gain seen within the first 6 months of treatment [5]. A second study comparing changes in weight following treatment of both hypothyroidism and hyperthyroidism also reported weight gain after treatment for GD early in follow up, though the exact time frame was not specified [11].

Unsurprisingly, a history of weight loss before RAI was associated with greater weight gain at follow up in our population, though this relationship was not present in the sub-analysis. However, prepubertal patients in our sub-analysis did have a greater ∆BMI SDS. This is in line with another study that observed greater increases in BMI after treatment for GD in children under 11 years compared with older children [5]. It is unknown whether this relationship is a result of age or of the metabolic changes that occur during puberty. No other factors were found to clearly define higher-risk groups for weight gain in our population. All patients receiving RAI for GD should be considered at high risk for weight gain and counseled accordingly.

Although our study was focused on the effects of RAI therapy, 21% of patients had previously received ATDs and experienced no difference in ∆BMI SDS at one year compared with those who had not. Most patients treated with ATDs were not seen at our institution at their initial presentation with GD, and thus their pre-treatment BMIs were not available. However, previous research has suggested that the use of sequential treatment modalities in adults can result in continued weight gain compared with definitive treatment as a first line approach [8]. This question would be interesting to investigate in pediatric patients as well. If corroborated, this may be further reason to advocate for earlier definitive treatment rather than pursuing medical therapy which only rarely results in permanent remission in children and adolescents and carries the potential for rare but serious side effects [12].

While TTE was negatively correlated with ∆BMI SDS in our subanalysis, the weakness of this association suggests that other factors should also be considered. Though some studies in adults show differences in BMI outcome by treatment modality [8, 9], this has not been demonstrated consistently, and it is unknown whether these expectations can be extended to children. We found similar outcomes in our population when compared to reports from other centers [5].

To our knowledge, our study represents the largest cohort of pediatric patients in whom weight gain following treatment for GD is investigated, and the only one in which all patients were treated with RAI. Limitations include its retrospective nature as well as the fact that data documenting the precise TTE was only available in 81 of our patients. An additional weakness is that we did not have information regarding our patients’ BMIs prior to the development of GD. However, nearly 40% of our patients were either overweight or obese at one year, a rate above national BMI SDS data in youth 2–19 years of age [13].

## Conclusions

In conclusion, we observed a striking and nearly universal increase in BMI SDS one year after RAI treatment for GD resulting in a doubling in the number of overweight and a tripling in the number of obese patients, placing them at higher risk for adverse health consequences over time. Future studies should focus on targeted interventions aimed at attenuating the rate of weight gain in children and adolescents undergoing treatment for GD.

## Abbreviations

∆BMI SDS: Change in BMI standard deviation score
ATDs: Anti-thyroid drugs
DS: Down syndrome
GD: Graves Disease
RAI: Radioactive iodine
TTE: Time to euthyroidism
